# Development and Application of a Novel Tsunami Monitoring System Based on Submerged Mooring

**DOI:** 10.3390/s24186048

**Published:** 2024-09-19

**Authors:** Baocheng Zhou, Xinwen Zhang, Xiaozheng Wan, Tongmu Liu, Yuqiang Liu, Hua Huang, Jing Chen

**Affiliations:** 1 South China Sea Marine Survey Center, Ministry of Natural Resources, Guangzhou 510300, China; 18312022312@163.com (B.Z.); xinwenzh@foxmail.com (X.Z.); 13580451879@139.com (Y.L.); hhaostos@126.com (H.H.); chenjing0919@126.com (J.C.); 2 Key Laboratory of Marine Environmental Survey Technology and Application, Ministry of Natural Resources, Guangzhou 510300, China; 3 School of Marine Technology and Science, Qilu University of Technology (Shandong Academy of Sciences), Qingdao 266100, China; 18661817997@163.com; 4 Institute of Oceanographic Instrumentation, Shandong Academy of Sciences, Qingdao 266100, China

**Keywords:** tsunami monitoring, submersible mooring system, real-time data transmission, inductive coupling

## Abstract

Real-time data transmission and reliable operation are essential for a tsunami monitoring system to provide effective data. In this study, a novel real-time tsunami monitoring system is designed based on a submersible mooring system. This system is equipped with a data acquisition and tsunami wave identification algorithm, which can collect the measured data of the pressure sensor and detect a tsunami wave in real time. It adopts the combination design of underwater inductive coupling transmission and a redundant BeiDou communication device on the water surface to ensure the reliability of real-time data transmission. Compared with traditional tsunami monitoring buoys, it has the advantages of reliable communication, good concealment, high security, and convenient deployment, recovery, and maintenance. The results of laboratory and sea tests show that the system has high reliability of data transmission, stable overall operation of the system, and good application prospects in the field of real-time tsunami monitoring and early warning.

## 1. Introduction

Tsunamis are gravitational water waves in the ocean, mainly caused by subduction zone thrust earthquakes, submarine landslides, volcanic eruptions, etc., which cause fractures in the seabed strata and cause fluctuations in the seawater. When a tsunami propagates in open waters, its wavelength is long and its height is small, allowing it to travel long distances across the ocean without significant energy loss. When a tsunami wave enters the continental shelf, it causes significant damage to coastal residents and facilities due to a sharp shallower water depth, shorter wavelength, and a sudden increase in wave height [1,2,3]. Since the 20th century, there have been a total of seven major tsunamis worldwide; among these, the most severe tsunami was caused by the 2004 Sumatra earthquake in Indonesia, resulting in huge casualties and property damage to coastal countries [4,5]. Countries around the world have also begun to strengthen monitoring and early warning of tsunamis, improve their early response capabilities, and reduce personnel and property damage [6].

According to research, establishing an effective tsunami monitoring system to real-time transmit tsunami wave data can enable timely and accurate predictions of the arrival time and wave height of tsunamis when they propagate to coastal areas. Its key data can effectively ensure the accuracy of tsunami warnings and reduce the disasters caused by tsunamis. Based on the characteristics of tsunamis, real-time tsunami monitoring systems typically use buoys or submerged beacon structures. Water level monitoring sensors are deployed at anchor positions near the seabed to invert real-time tide level changes. By comparing the difference between actual and predicted tide levels, tsunami waves are determined, and tsunami data are uploaded to surface communication buoys. Then, these data are transmitted to ground observation stations through satellites to monitor real-time changes in sea level elevation related to tsunamis [7]. Sharadha studied the use of autonomous underwater robots to monitor changes in the underwater environment and provide warnings for underwater earthquakes and tsunamis, but there is the problem of real-time and reliable data transmission [8]. Researchers have also constructed earthquake monitoring networks, as described in the literature [9]. By deploying multiple tide gauges in Morocco to form an observation array and transmitting real-time data through satellites, tsunami observation and warning were achieved [10]. The National Oceanic and Atmospheric Administration (NOAA) of the United States has deployed multiple tsunami buoys in the deep ocean, known as DART (Deep Ocean Assessment and Reporting of Tsunami) systems, which can monitor tsunami waves in real time and transmit the data back to shore station data centers. Researchers can use tsunami waves to infer their source, and issue tsunami warning information in a timely manner, which have good application effects. However, networked array systems face high costs and difficulty in maintaining underwater equipment [11]. In addition to using tide level observation technology, researchers have also conducted research on tsunami monitoring systems based on fiber-optic interference technology. For example, the State Oceanic Administration of China has deployed a tsunami monitoring system equipped with fiber-optic interference water level monitoring instruments, with a measurement accuracy of ±1 cm, which has achieved good applications. However, the detection range of this type of fiber optic is 0–110 m, which is difficult to adapt to early warning of tsunamis in deep-sea environments [12].

Therefore, although tsunami monitoring systems have achieved good application results in tsunami warning at present, most of them have high construction and operation costs, and the reliability of real-time transmission of deep-sea data is difficult to guarantee [13,14,15,16,17,18]. Therefore, this study designs a new tsunami monitoring system based on a submerged beacon platform. The system deploys data acquisition and processing algorithms in a data acquisition and control system, adopts a highly reliable redundant communication scheme to achieve reliable transmission of tsunami data, and conducts sea trials in the South China Sea to verify the effectiveness and feasibility of the system designed in this study.

## 2. Overall System Design

**Figure 1:**
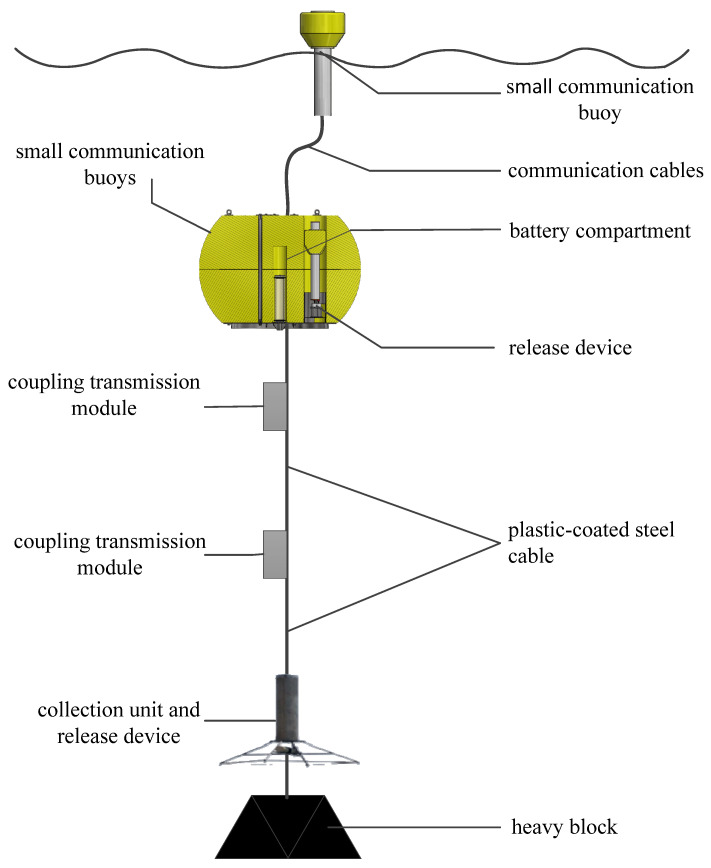
Overall design (reconstruction) of coupled transmission tsunami buoy system.

The highly reliable tsunami real-time monitoring system adopts a submarine anchor system structure design. It measures deep-sea seabed pressure data in real time through an underwater pressure sensor unit. The underwater data collection unit can calculate the corresponding tidal level value during the corresponding period based on the pressure data and detect tsunami waves. The overall system design is shown in Figure 1. According to the research, the anchor system structure of a submersible can improve the safety of the system, reduce the risk of human damage, and ensure the long-term in-place operation of the system [19,20].

The highly reliable tsunami real-time monitoring system consists of a data acquisition and processing subsystem, a mooring and release subsystem, a data transmission subsystem, and a shore station data-receiving subsystem. The following is a detailed introduction to each subsystem.

The data acquisition and processing subsystem is the core function of the tsunami real-time monitoring system, mainly realizing functions such as pressure data acquisition, raw data processing, tsunami wave determination, data communication control, and feedback. This subsystem includes hardware modules such as pressure sensors and data collectors. The pressure sensors collect pressure data in real-time and transmit them to the data collector through coupling transmission. At the data collector end, tidal level values are calculated, and tsunami waves are determined. After a tsunami wave propagation event is identified, a tsunami alarm is triggered, and the original data within the corresponding time period are packaged and transmitted back to the shore station data-receiving subsystem.

The mooring and release subsystem is the basic platform for ensuring the in situ operation and real-time data transmission of the tsunami monitoring system. It connects various hardware modules in the ocean through an anchoring structure and is designed with an automatic release communication buoy, which can ensure the reliable ejection of the water-surface-communication buoy. This subsystem mainly includes surface communication buoys, communication cables, underwater buoys (equipped with surface communication buoy devices and release devices), data collectors, plastic-coated steel cables (equipped with coupling transmission devices), underwater units (equipped with pressure sensors and release devices), and anchoring weights.

The data transmission subsystem adopts multiple redundant communication methods to achieve real-time data transmission back to the shore station data-receiving subsystem, which is essential to achieving real-time tsunami warnings. This subsystem consists of coupling transmission modules, plastic-coated steel cables, communication buoys, and satellite transceiver devices. The real-time measurement data of the pressure sensor on the seabed are transmitted to the data collector through a coupled transmission module. If the data collector recognizes a tsunami wave, it triggers a tsunami warning. The mooring and release subsystem will use surface communication buoys to send the data to the shore station data-receiving subsystem via satellite [21].

The shore station data-receiving subsystem adopts a server/client development mode. The server side is responsible for buoy configuration management, data communication reception and storage, and tsunami data status and alarms. The client side realizes multi-user data inquiry, data analysis, and buoy status and tsunami status display management operations through network access and data interface query. The system mainly implements the following functions: (1) communication function: real-time reception of BeiDou satellite communication data sent by surface communication buoys and verification of the messages; (2) storage and query functions: these realize the storage of various parameters such as the status, position, and tsunami monitoring pressure values of the parsed buoys and provide efficient and convenient database access services; (3) display function: this displays the status, location, and data information of the tsunami monitoring system, as well as the status and pressure monitoring values of the tsunami units.

## 3. Key Technologies

The tsunami monitoring system proposed in this article uses its anchoring system to deploy pressure sensors near the seabed to record real-time seabed pressure data. When a tsunami wave passes through the sea surface where the pressure sensor is located, it will cause a change in wave height, which, in turn, will cause a change in seabed pressure. According to the recorded historical pressure, the sea surface height can be inferred as the height of the tsunami wave. When confirming the tsunami wave, the original pressure data are sent to the remote data center on the ground through satellite communication buoys for real-time tsunami warning analysis [22].

### 3.1. Data Collection and Tsunami Wave Identification Methods

The tsunami monitoring system completes pressure data collection, tsunami wave identification, and communication control through data acquisition units deployed underwater. After the system is deployed, it can be divided into deployment mode, standard mode, and tsunami alarm mode according to different data processing methods. The system is in deployment mode for 3 h after entering the water, at which point the pressure data have been stably obtained for a certain period of time; the standard mode mainly realizes the collection of pressure data and the determination of tsunami waves. The main implementation method is used to identify tsunami waves by inverting tidal level data based on historical pressure data and measured data and comparing them. The system status information is transmitted back every hour. The tsunami alarm mode detects tsunami waves and sends the original data back to the remote data center. The following is a detailed introduction to the method of determining tsunami waves and the data transmission scheme:

(1) Use pressure sensors to obtain deep-sea seabed pressure data for a period of time (reaching data stability within 3 h after deployment), in order to obtain the corresponding tidal level values for the time period.

(2) Perform real-time quality control processing of raw data on the data collector end. Firstly, coarse-grained removal of formatting errors and data exceeding the threshold in the original data is performed, and further detection is carried out using the three-sigma method within a 1 h data range. Discrete independent data are removed. Use interpolation polynomials to fit astronomical tidal waveforms, collect data at different times, use time as the interpolation point on the *x*-axis, and predict the tidal level value H’ (t’) for the next time t’.

$$H’(t’)=\sum_{i=0}^{3}w(i)\cdot H_{i}^{*}(t-p/2-i\Delta t)$$

**Figure 2:**
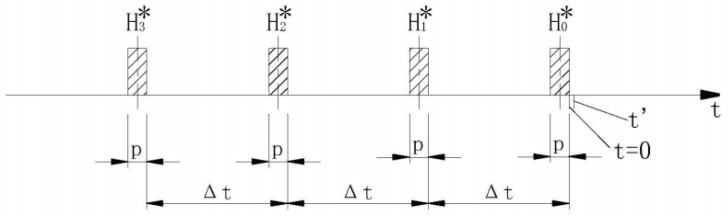
Schematic diagram of tsunami processing algorithm.

The tsunami processing algorithm is shown in Figure 2, where t is the current time, t’ is the predicted time (data 5.25 min after the current time t), p is the duration of the arithmetic mean method (10 min), and Δt is the interval time (1 h) between two adjacent interpolation points. Set the end time of monitoring to the current time t = 0. Average the pressure data within the first p minutes before the current time t, Δt, 2Δt, and 3Δt to obtain four tidal levels: $H_{0}^{*}$, $H_{1}^{*}$, $H_{2}^{*}$, and $H_{3}^{*}$.

**Figure 3:**
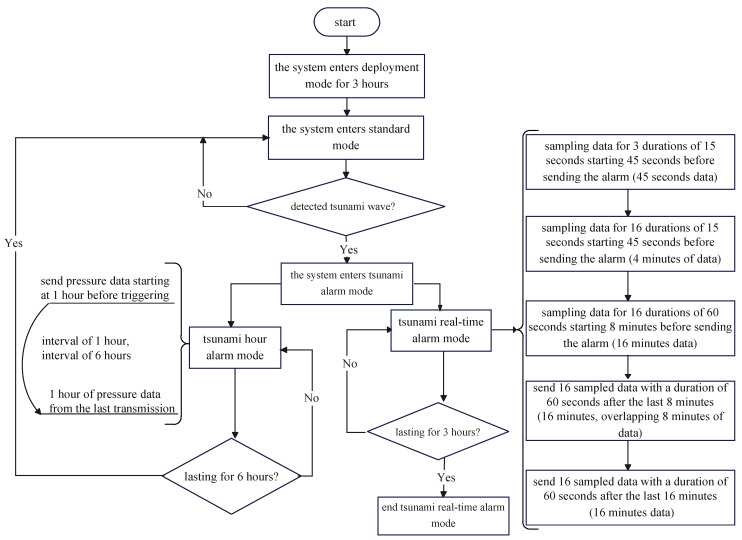
Data collection and processing in the tsunami monitoring system.

(3) Measure the deep-sea seabed pressure data at time t’ to obtain the measured tidal level value at time t’.

(4) Calculate the difference between the measured tidal level value and the predicted tidal level value. Based on continuous multiple difference data, we determine whether there is tsunami wave propagation. If tsunami wave transmission is detected, activate the tsunami warning mode for feedback alarm. The system workflow is shown in Figure 3.

### 3.2. High-Reliability Data Transmission Design

The tsunami monitoring system mainly provides real-time data support for tsunami warning, and reliable data transmission methods are key to ensuring the real-time aspect and accuracy of tsunami warning. The real-time transmission path of tsunami monitoring system data is as follows: pressure sensor → underwater data collector → surface satellite communication equipment → remote data center. This article presents a design which combines underwater induction coupled transmission and a redundant satellite communication device on the water surface to ensure the reliability of real-time data transmission.

#### 3.2.1. Inductively Coupled Transmission Design

**Figure 4:**
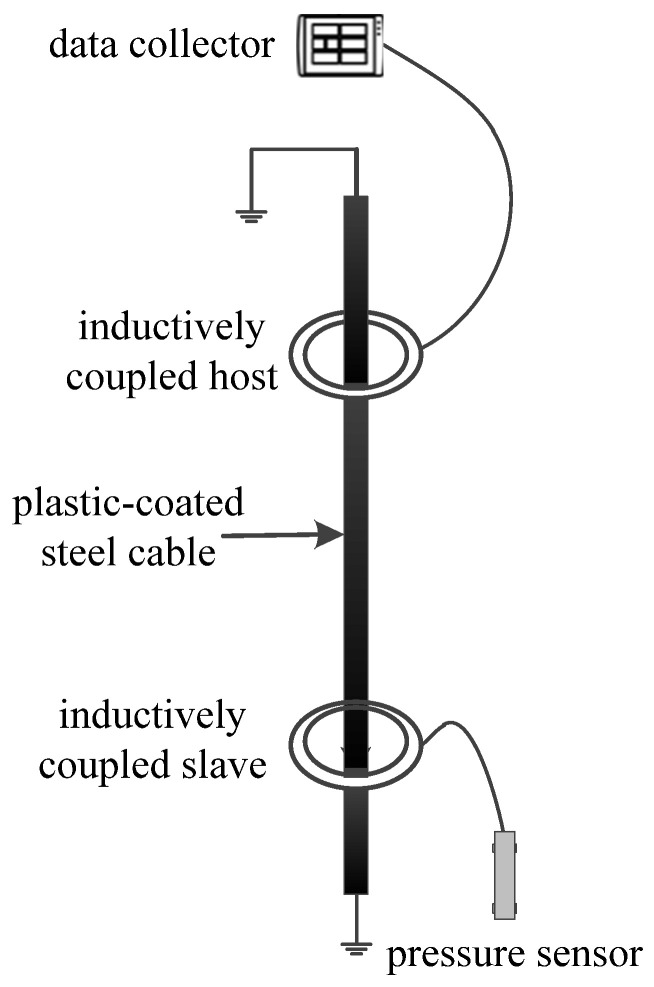
Schematic diagram of inductive coupling transmission.

Commonly used underwater data transmission technologies currently include cable transmission and wireless transmission. Cable transmission often uses multi-core watertight cables to connect sensors for communication. However, tsunami monitoring systems are located in deep water areas with long anchoring structures, which have the disadvantages of signal strength attenuation with distance and cable damage; wireless transmission technology includes laser communication, underwater acoustic communication, and inductive coupling communication, of which laser communication has strong directionality [23,24]. Factors such as water quality and plankton often affect communication effectiveness, limiting its application range [25,26]; underwater acoustic communication utilizes sound waves for underwater communication. The fluctuation and multipath effects of the underwater acoustic channel result in a low propagation rate, large delay, high bit error rate, and high power consumption of transceivers, which is not conducive to the long-term operation of tsunami monitoring systems. Electromagnetic induction coupling communication technology relies on small-sized coupling coils to induce magnetic field components for communication. Its magnetic induction signal propagates at the speed of light in the transmission medium, which has the advantages of a small transmission delay, stable channel, and high reliability and has good application prospects [27,28]. This article uses electromagnetic induction coupling technology for underwater transmission to achieve real-time transmission of pressure sensor data to the data collector, improving the reliability of data transmission. A schematic diagram of induction coupling transmission is shown in Figure 4.

#### 3.2.2. Redundant Design of Water Surface Communication

The air link for data transmission through satellite communication devices already has mature communication technologies and applications, such as the Iridium communications service, maritime satellites, BeiDou short messages, and Tiantong satellites, to complete data transmission. This article uses the BeiDou satellite for data transmission. In the data transmission subsystem, the BeiDou communication antenna is installed inside the surface communication buoy and connected to the underwater unit through communication cables. The data collector is integrated into the underwater unit and has a certain computing capability. It can receive real-time data collected by the water level gauge and calculate pressure data. When a tsunami wave is detected, the device sends the data to the BeiDou satellite antenna through a communication cable and sends it back to the remote data center.

**Figure 5:**
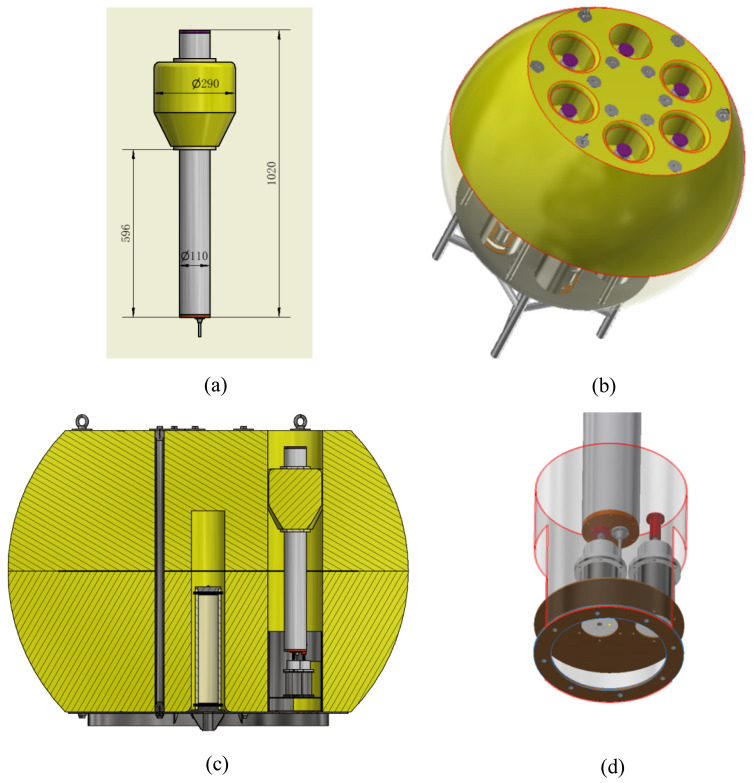
Design of underwater unit and communication buoy structure. (**a**) Small communication buoy, (**b**) Underwater unit, (**c**) Installation structure of small communication buoys, (**d**) Release device.

To ensure the reliability of tsunami data transmission, the small communication buoy adopts a redundant design. The underwater unit can carry six small communication buoys as backups for the BeiDou satellite antenna. The design of the small surface-communication buoy is shown in Figure 5a, and the underwater unit is shown in Figure 5b,c. After the system is deployed, the first communication buoy is released onto the sea surface for data communication. The other five communication buoys are stored inside the underwater unit and fixed to the installation structure of the buoy through a release device. The installation structure of the buoy is fixed to the bottom of the underwater unit through bolts. The release device of the underwater buoy is shown in Figure 5d.

**Figure 6:**
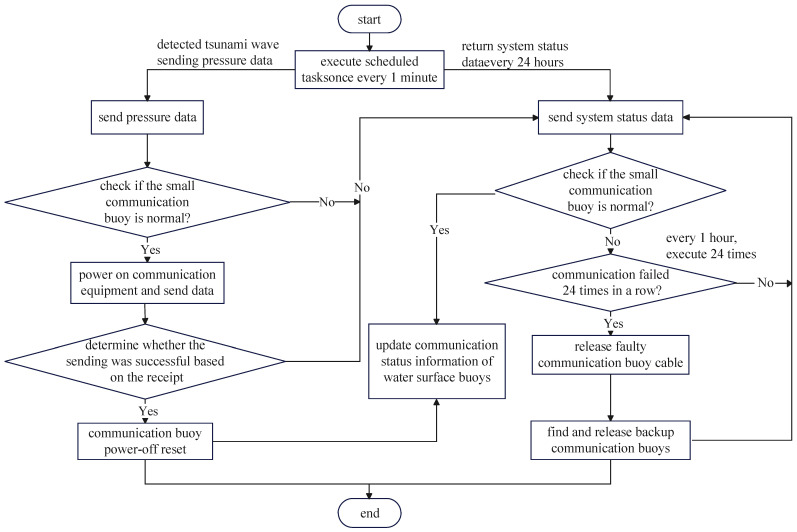
Release device control mechanism process.

The release of the communication buoys is controlled by a data collector equipped with a data acquisition and processing subsystem inside the underwater unit. When the collector loses contact with the communication buoy, communication testing or the release of new sea surface buoys will be carried out to ensure the reliable transmission of tsunami data. The release mechanism process of the small surface-communication buoy is shown in Figure 6.

## 4. Sea Trial Verification and Result Analysis

### 4.1. Prototype System Construction

**Figure 7:**
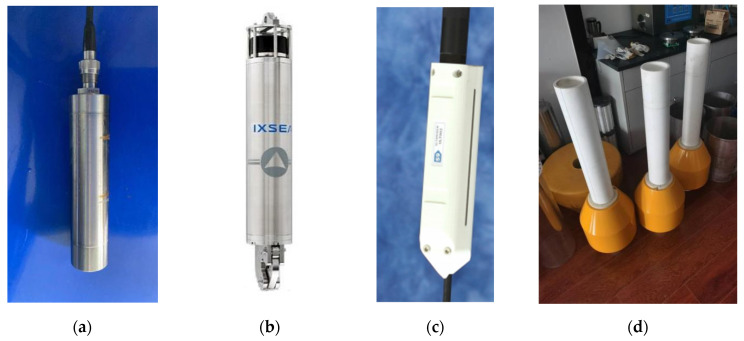

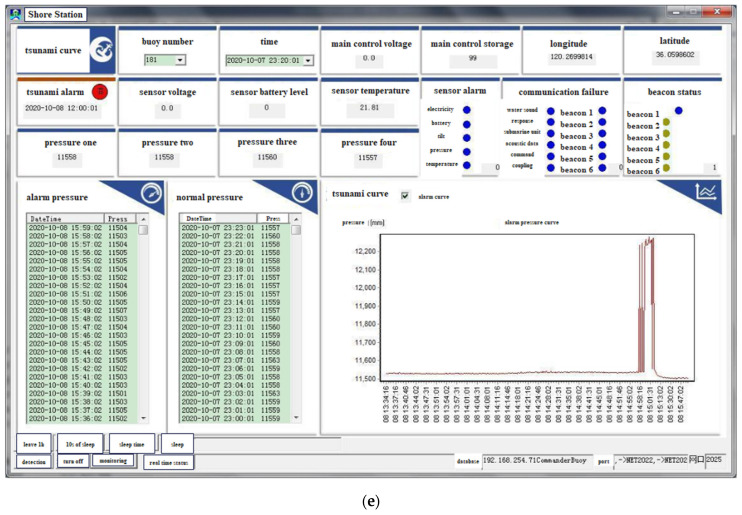
Physical diagrams of the prototype system. (**a**) Pressure sensor, (**b**) Acoustic release device, (**c**) Inductive coupling device, (**d**) Small water-surface-communication buoy, (**e**) Shore station data-receiving subsystem.

To verify the effectiveness and feasibility of the system design in this article, a prototype system was built, and sea trials were conducted. The prototype system adopts the Paroscientific 8CB7000-I pressure sensor (Washington, UT, USA), the IXSEA-Oceano 5000 acoustic release device (Paris, France), and the Soundnine S9 induction coupling device (Washington, UT, USA). The satellite communication device integrates twelve 60-second-frequency BeiDou card (Beijing, China), which can launch data packets of 1 KB immediately to meet the real-time transmission requirements of tsunami raw data. The main configuration of the system is shown in Table 1, and the actual system is shown in Figure 7.

### 4.2. Laboratory Copying Situation

A laboratory copying machine is mainly used to verify the working mechanism of the entire system and the stability and reliability of the system operation. The main verification content includes the success rate of data transmission and reception, the switching of the working mode of the tsunami monitoring system, and the release of the small water-surface-communication buoy device.

**Figure 8:**
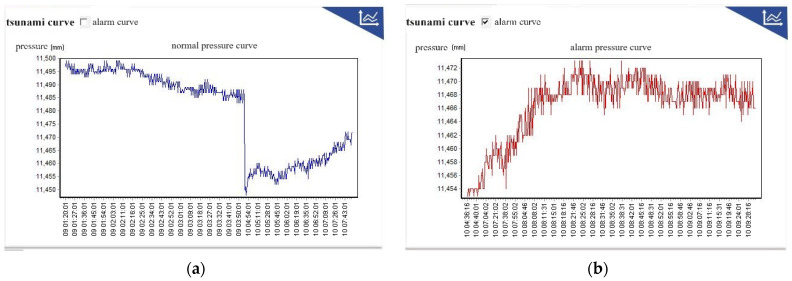
Normal pressure and alarm pressure curves simulated by laboratory copying machine. (**a**) Normal pressure curve, (**b**) Alarm pressure curve.

The laboratory copying process goes from deployment mode to standard mode, from standard mode to alarm mode, and from alarm mode to standard mode. The success rate of data transmission and reception during the mode switching process is 100%, and several working mode switches are successfully simulated without any abnormalities in the working mechanism. The data reception is detailed in Table 2, and the pressure data curve is shown in Figure 8.

**Figure 9:**
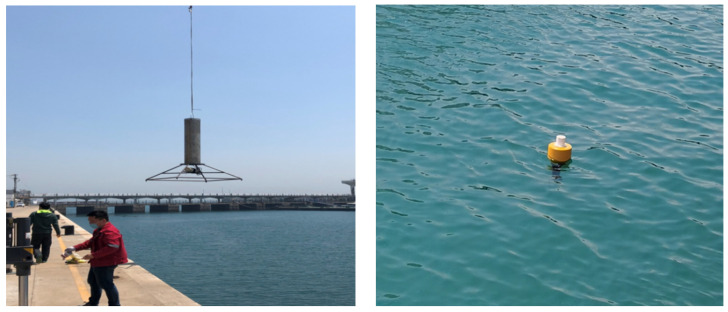
Release device shore test.

The release test on the sea surface buoy ejection device was conducted at the dock. After testing, it was found that the underwater release performance of the small water-surface-communication buoy release device was good, and the communication cable had slight positive buoyancy and no entanglement after being ejected. The shore test process of the release device is shown in Figure 9.

### 4.3. Sea Trials in the South China Sea

To verify the actual performance and reliability of the tsunami real-time monitoring system proposed in this article, a complete deployment and testing of the system was conducted on 25 September 2020 in the 54-meter-deep sea area of the South China Sea to verify the overall reliability of the tsunami real-time monitoring system function.

The overall deployment sequence of the system is as follows: first, place the underwater unit in the water, then place the parallel release device in the water, fix its lower-end under force on the mooring pile, release it with a hook, and slowly place the Kevlar cable in the water until the weight block is in place. Use an A-frame to lift the weight block into the water, release the hook, and complete the system deployment.

**Figure 10:**
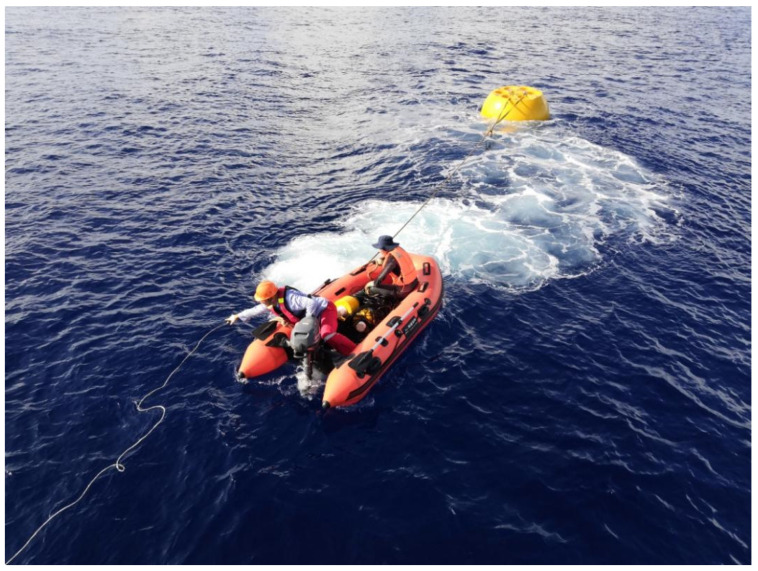
Small-boat recovery underwater unit.

The sea trial began on the morning of 25 September 2020. At 9:52, the entire system was powered on, and the data reception was normal. The deployment was completed at 10:30. At 16:30, the system was released to the water surface through the deck unit and was towed to the side of the boat by another small boat (Figure 10). It was successfully recovered using the onboard A-frame. After the system recycling, upon reviewing the original data, it was found that after the deployment mode ended, it successfully entered the standard mode at 13:30, and the pressure data were collected completely. The system status information was transmitted back to the shore station data center every hour. After entering standard mode, the first small water-surface-communication buoy was successfully released, and 10 min later, the second small buoy was successfully released (the simulation test showed that the first small buoy was damaged). The redundant design of water-surface-communication was running well. Table 3 shows the partial data of the sea trial, and the data results indicate that the system runs stably and the data are good.

## 5. Discussion and Conclusions

This article presents a novel system for tsunami monitoring using a submerged mooring system equipped with pressure sensors and real-time data transmission capabilities. The system collects real-time pressure data from pressure sensors near the seabed for tsunami wave detection and divides the underwater operation of the system into deployment mode, standard mode, and tsunami alarm mode according to different data processing methods. The results of the laboratory and sea tests show that the overall operation of the system is stable, and the data acquisition and tsunami wave recognition algorithms carried on it operate stably, with a data acquisition rate of 100%. The combination design of underwater induction coupling transmission and water surface redundant BeiDou communication device used in the system performed well. During the testing period, the data reception rate of the shore station data-receiving subsystem reached 100%, which verifies the feasibility of the proposed method. The data collection rate and data reception rate both reached 100%, which can ensure timely acquisition of data during the tsunami and release warning information as soon as possible, ensuring personnel safety and avoiding property damage.

Compared with the novel tsunami monitoring buoys, traditional buoys are seriously affected by bad sea conditions, ship collision, human damage, and biological attachment because they float on the sea surface. The submerged buoy-type tsunami monitoring system designed in this article is submerged underwater and has good concealment and safety. On the other hand, the communication method of inductive coupling adopted in this study is more stable and reliable compared to the acoustic communication of traditional buoys. In addition, submerged beacons are easier to deploy, recover, and operate and maintain than buoys. During sea trials, the test data show that the system design is safe and reliable, runs stably, and has the characteristics of convenient and reliable deployment, recycling, and operation, enriching the technical means in the field of tsunami monitoring.

In future work, to further validate the stability and reliability of the system, the novel tsunami monitoring system designed herein will be validated at sea over a long period of time and compared with traditional tsunami buoy and seismic station measurements. In addition, sensors such as temperature, salinity, and seismometers can be added to moorings and submarine installations to obtain more data for ocean research while monitoring tsunamis.

## Figures and Tables

**Table 1:** Prototype system hardware configuration.

Number	Name	Model	Main Parameter
1	Pressure sensor	Paroscientific 8CB7000-I	Working water depth ≤ 7000 m; accuracy: 0.01% FS
2	Acoustic release device	IXSEA-Oceano Oceano5000	Working water depth ≤ 6000 m; Communication distance ≤ 10 km
3	Anchor weight block	Customization	Anchor block weight: 2000 kg
4	Inductive coupling device	Soundnine/S9	Working water depth ≤ 4500 m
5	Underwater unit	Customization	Diameter of 2 m; height of 1.4 m; reserve buoyancy: 3097 kg; equipped with 6 release devices
6	Communication device	BeiDou Second Generation	Integrate twelve 60-ssecond-frequency BeiDou civilian cards with a communication capacity of 1 KB/min
7	Plastic-coated steel cable	Customization	Outer diameter 8.4 mm; inner diameter 6.4 mm; tensile strength ≥ 2 kg
8	Data collector	Customized development board	Equipped with 32 GB storage and 8 RS232 interfaces; the operating power consumption is approximately ≤1 W; and the sleep power consumption is ≤200 mW

**Table 2:** Prototype system copying and data transmission situation.

Working Mode	Number of Actually Sent Data Packets	Actual Number of Received Data Packets	Data Reception Success Rate
Layout mode (lasting for actual 3 h)	47 packets	47 packets	100%
Alarm mode (actual duration of 6 h)	53 packets	53 packets	100%
Standard mode (actual duration of 9 h)	9 packets	9 packets	100%

**Table 3:** Partial data from sea trials in South China Sea.

BuoyID	Mode	DateTime	AlarmDateTime	PressMM
181	2	25 September 2020 15:03	25 September 2020 15:00	112,107
181	2	25 September 2020 15:03	25 September 2020 15:00	112,106
181	2	25 September 2020 15:03	25 September 2020 15:00	112,106
181	2	25 September 2020 15:04	25 September 2020 15:00	112,102
181	2	25 September 2020 15:04	25 September 2020 15:00	112,098
181	2	25 September 2020 15:04	25 September 2020 15:00	112,100
181	2	25 September 2020 15:04	25 September 2020 15:00	112,102
181	2	25 September 2020 15:05	25 September 2020 15:00	112,098
181	2	25 September 2020 15:05	25 September 2020 15:00	112,097
181	2	25 September 2020 15:05	25 September 2020 15:00	112,103
181	2	25 September 2020 15:05	25 September 2020 15:00	112,098
181	2	25 September 2020 15:06	25 September 2020 15:00	112,103
181	2	25 September 2020 15:06	25 September 2020 15:00	112,098
181	2	25 September 2020 15:06	25 September 2020 15:00	112,097
181	2	25 September 2020 15:06	25 September 2020 15:00	112,106
181	2	25 September 2020 15:07	25 September 2020 15:00	112,104
181	2	25 September 2020 15:07	25 September 2020 15:00	112,106
181	2	25 September 2020 15:07	25 September 2020 15:00	112,111
181	2	25 September 2020 15:07	25 September 2020 15:00	112,109
181	2	25 September 2020 15:08	25 September 2020 15:00	112,114
181	2	25 September 2020 15:08	25 September 2020 15:00	112,113
181	2	25 September 2020 15:08	25 September 2020 15:00	112,124
181	2	25 September 2020 15:08	25 September 2020 15:00	112,126
181	2	25 September 2020 15:09	25 September 2020 15:00	112,125
181	2	25 September 2020 15:09	25 September 2020 15:00	112,131
181	2	25 September 2020 15:09	25 September 2020 15:00	112,136
181	2	25 September 2020 15:09	25 September 2020 15:00	112,135
181	2	25 September 2020 15:10	25 September 2020 15:00	112,138
181	2	25 September 2020 15:10	25 September 2020 15:00	112,143
181	2	25 September 2020 15:10	25 September 2020 15:00	112,139
181	2	25 September 2020 15:10	25 September 2020 15:00	112,136
181	2	25 September 2020 15:11	25 September 2020 15:00	112,138

## Data Availability

Access to the data will be considered upon request by the authors.
